# A dZnONPs Enhanced Hybrid Injectable Photocrosslinked Hydrogel for Infected Wounds Treatment

**DOI:** 10.3390/gels8080463

**Published:** 2022-07-24

**Authors:** Yao Chen, Yu Xiang, Tonghe Zhu, Sihao Chen, Juan Du, Jiajia Luo, Xiaoyu Yan

**Affiliations:** 1 School of Chemistry and Chemical Engineering, Shanghai Engineering Research Center of Pharmaceutical Intelligent Equipment, Shanghai Frontiers Science Research Center for Druggability of Cardiovascular Non-Coding RNA, Institute for Frontier Medical Technology, Shanghai University of Engineering Science, 333 Longteng Rd., Shanghai 201620, China; m040119408@sues.edu.cn (Y.C.); chensh@sues.edu.cn (S.C.); bairuochen12@163.com (J.D.); jiajialuo1108@163.com (J.L.); 2 Department of Sports Medicine, Department of Orthopedics, Shanghai Jiao Tong University Affiliated Sixth People’s Hospital, 600 Yishan Rd., Shanghai 200233, China; knight-errant@sjtu.edu.cn

**Keywords:** hydrogel, injectable, infected wounds, antibacteria, microvascularization

## Abstract

Chronic wounds caused by related diseases such as ischemia, diabetes, and venous stasis are often hard to manage, mainly because of their susceptibility to infection and the lack of healing-promoting growth factors. Functional hydrogel is a promising material for wound treatment due to its regulable swelling rate and its ability to absorb wound exudate, which can keep the wound isolated from the outside world to prevent infection. In this study, a photocrosslinked physicochemical double-network hydrogel with injectable, antibacterial, and excellent mechanical properties was prepared. The dZnONPs enhanced hybrid injectable photocrosslinked double-network hydrogel (Ebs@dZnONPs/HGT) was synthetized starting from acylated hyaluronic acid and tannic acid via free radical reaction and hydrogen bonding, following doped with ebselen (Ebs) loaded dendritic zinc oxide nanoparticles (dZnONPs) to prepare the Ebs@dZnONPs/HGT hydrogel. The physicochemical characterization confirmed that the Ebs@dZnONPs/HGT hydrogel had excellent mechanical properties, hydrophilicity, and injectable properties, and could fit irregular wounds well. In vitro experiments revealed that the Ebs@dZnONPs/HGT hydrogel presented credible cytocompatibility and prominent antibacterial activity against Escherichia coli (*E. coli*) and Staphylococcus aureus (*S. aureus*). In vivo experiments further demonstrated that the Ebs@dZnONPs/HGT hydrogel had excellent biosafety and could improve re-epithelialization in the wound area, thus significantly accelerating wound healing.

## 1. Introduction

The skin is the biggest organ in the human body. It functions as the first barrier to the physical environment, preventing excessive water loss and protecting against harmful substances and pathogenic microorganisms [1,2]. Skin injuries are caused by multiple factors, such as injuries, medical treatments, and diseases [3]. Due to an insufficient physical barrier and exposed subcutaneous tissue, skin defects are susceptible to bacterial infections. *S. aureus* and *E. coli*, for example, are the most common and important pathogens responsible for wound infections [4,5]. Furthermore, wound infection may be accompanied by soft tissue defects such as muscle and fascia, resulting in irregular outward appearance and depth of the wound. The healing of the infected wound is slow, increasing the financial burden and leading to significant health threats [2]. Therefore, for the repair of infected wounds, it is critical to develop a dressing that can not only conform well to the wound but also have powerful antibacterial properties.

Some wound dressings, such as hemostatic sponges [6], foams [7], and fibrous membranes [8], have been designed and proposed in recent years. Among them, hydrogel is becoming one of the most competitive wound dressings due to its unique performance such as high water content, biocompatibility, degradability, drug delivery, and the 3D porous structure similar to extracellular matrix (ECM) [9,10]. Both sponge and fibrous membrane are difficult to completely fit the wound surface and have application limitations. Hydrogel can be injected into the wound area for a perfect fit [11], and photosensitive hydrogel can be transformed from solution to gel. Hyaluronic acid (HA) is a disaccharide-based straight-chain polymer with excellent biodegradability and rheological properties. Moreover, it contains free hydroxyl and carboxyl groups that can be modified under mild conditions [12,13]. For instance, after introducing the photo-sensitive group into the branched chain of HA, radical polymerization occurs under the action of ultraviolet light to realize the transition from solution to gel [14,15,16]. As one of the main compositions of human tissue’s ECM, HA can be broken down into glucosamine and absorbed by the human body [17,18], indicating that it is biocompatible.

Ebselen (Ebs) is a promising anti-infective agent that has potent antimicrobial activities against many kinds of bacteria like *Helicobacter pylori*, *S. aureus*, *Mycobacterium tuberculosis*, and *Bacillus anthracis* [19]. Ebs inhibits the activity of bacterial thioredoxin reductase (TrxR) competitively, disrupting bacterial redox homeostasis and causing the accumulation of reactive oxygen species (ROS) that ultimately cause bacteria death [20]. Due to structural and electron transport variations between human and bacterial TrxR, Ebs does not affect the survival of human cells and has excellent biosafety and biocompatibility [21,22,23]. On the other hand, Ebs possesses antioxidant and anti-inflammatory activity as a glutathione peroxidase analog, which can protect cells from oxidative injury, and reduce the toxicity and other side effects of many metal ions such as manganese, mercury, and cadmium [24]. ZnONPs is an inorganic antibacterial agent that can disrupt bacterial biofilms and has potent antibacterial properties for both gram-positive and gram-negative bacteria [25]. The antibacterial properties of ZnONPs are mainly due to oxidative stress produced by the increased ROS [26]. Although high concentrations of ZnO have a potent antibacterial effect, they can cause slight cytotoxicity, which is also linked to ROS production [27].

Based on the characteristics of ZnONPs and Ebs, we believe that combining the two will enhance the anti-infective effect while reducing ZnONP concentrations. At the same time, the antioxidant and detoxification properties of Ebs will reduce ZnONP-induced oxidative stress and cytotoxicity. Kalantari et al. [28] prepared dendritic mesoporous carbon nanoparticles (DMCNs) and proved that DMCNs have a high specific surface area and excellent adsorption capacity. Chu et al. [29] prepared dendritic fiber-type silica (KCC-1) with a large specific surface area and demonstrated that it has a high adsorption capacity. So we prepared dendritic zinc oxide nanoparticles (dZnONPs) that had more attachment sites. By loading Ebs onto dZnONPs, a more efficient and long-lasting antibacterial effect may be achieved. So far, few studies have applied Ebs in the treatment of infectious wounds. Whether combining Ebs and ZnONPs can improve antibacterial efficacy while reducing cytotoxicity has not been reported in the literature yet.

Here, we prepared a double-network hydrogel (Ebs@dZnONPs/HGT) with injectability, powerful antibacterial activity, and rapid photocrosslinking. HA was used as a substrate to introduce acrylate double bonds, which underwent a free radical reaction under UV light irradiation. Tannic acid (TA) was then added to form an intermolecular interaction, which eventually resulted in the formation of a physicochemical double-network hydrogel. When compared to the single-layer network, the double-layer network enhanced the hydrogel’s mechanical properties and fatigue resistance. The hydrogel could be injected easily and completely cover the wound, isolating the wound from the external environment. The hydrogel also had excellent water absorption, retention, biodegradability, and biocompatibility. We reported for the first time that the incorporation of Ebs@dZnONPs in hydrogels not only postponed the release of Ebs, but also had a more powerful antibacterial effect and better biological safety, and could accelerate the healing of an infected wound.

## 2. Results and Discussion

The skin is exposed constantly to injuries due to direct contact with the external environment, and the wound without a protective mechanism is susceptible to invasion by fungi, bacteria, or other microorganisms [30]. The microorganisms proliferate and produce harmful substances, causing the wound to fester and the epithelium to grow slowly or even stop growing [31]. At the same time, microbial infections can increase exudates around the wound site, which increases the local tension of the wound and even causes wound dehiscence. Therefore, it is urgent to design wound dressings with strong antibacterial properties to treat infected wounds and achieve effective wound healing.

HA is a natural polysaccharide present in almost all body tissues and fluids. It contains many active sites of carboxyl, hydroxyl, and amide, which can be chemically modified to expand its application [32]. The acrylate double bond with high activity was introduced into the branched chain of HA, and then a single-layer network structure was formed via UV-mediated free radical polymerization using the acylated HA [33]. Next, the double-network hydrogel was formed by intermolecular forces between TA with phenolic hydroxyl group and HA. Simple hydrogels have little effect on wound healing and cannot rapidly promote wound repair. Ebselen and ZnONPs have potential synergistic antibacterial and complementary effects. Therefore, the two were used in combination to improve the antibacterial properties while reducing the dosage of drugs.

**Figure 1:**
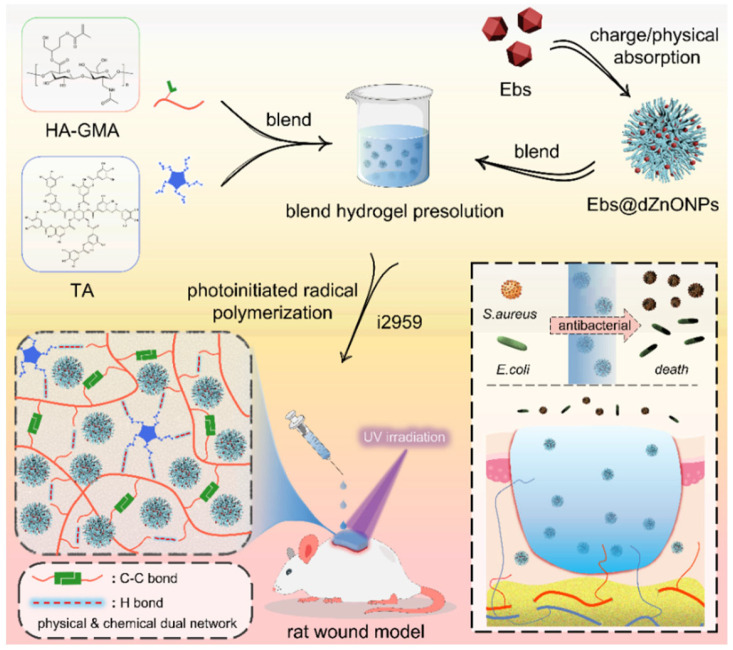
Schematic diagram of preparation of Ebs@dZnONPs/HGT hydrogel and its application in the treatment of infected wounds.

In this study, a double-network Ebs@dZnONPs/HGT hydrogel with strong antibacterial properties was designed and prepared. The hydrogel was injected into the infected wound and gelated by irradiation with UV light to achieve a complete fit to the wound. The combination of Ebs and ZnONPs killed bacteria around the wound, provided a suitable environment for the wound, and ultimately promoted the healing of infected wounds (Figure 1).

### 2.1. Dendritic Zinc Oxide Nanoparticles (dZnONPs)

**Figure 2:**
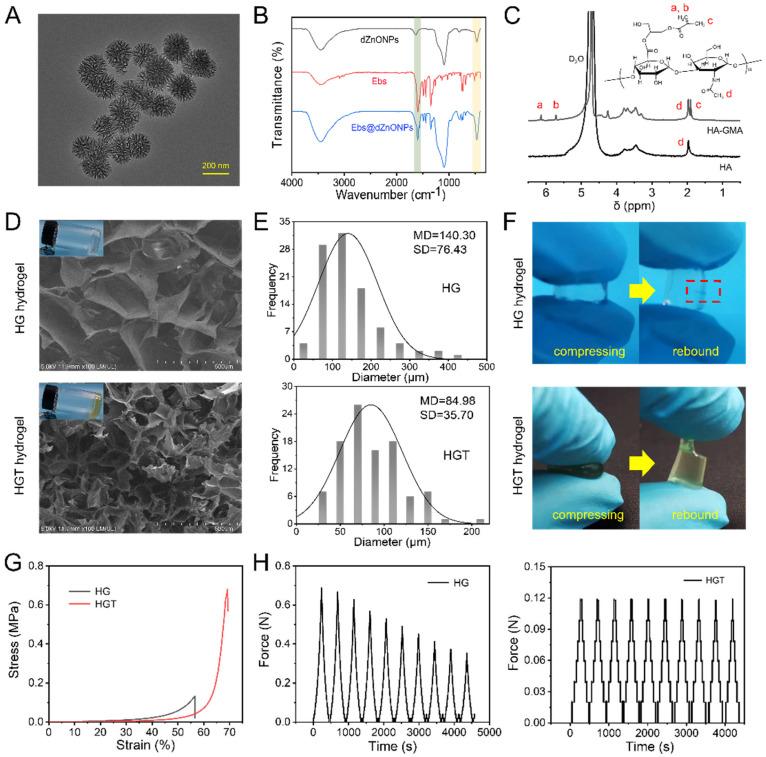
(**A**) TEM of dZnONPs; (**B**) FTIR of dZnONPs, Ebs, and Ebs@dZnONPs; (**C**) ^1^H-NMR of HA and HA-GMA; (**D**) Cross-sectional SEM of HG hydrogel and HGT hydrogel; (**E**) Pore size distribution of HG hydrogel and HGT hydrogel; (**F**) Mechanical macrographs of HG hydrogel and HGT hydrogel; (**G**) Single compressive stress-strain curves of HG hydrogel and HGT hydrogel; (**H**) Resilience-time diagram of HG hydrogel and HGT hydrogel (10 compression cycles).

To promote effective wound healing, existing studies suggest that the use of nanoparticles as drug carriers will have better release kinetics, especially dendritic nanoparticles which, due to their larger specific surface area, can confer more attachment sites for the drug [34,35]. In this study, dendritic zinc oxide nanoparticles were successfully prepared using a cationic surfactant as the template, ethyl orthosilicate as the silica source, NaOH as the catalyst and the organic solvent cyclohexane as the oil phase (Figure 2A), and the particle size distribution curves showed that the resulting dendritic spherical particles had a good particle size distribution (160 nm~180 nm) (Figure S1A).

In order to confirm whether the Ebs bound to the dZnONPs, they were characterized using Zeta potential analysis and FTIR spectroscopy. The results of Zeta potential showed that dZnONPs were negatively charged and Ebs were positively charged. Mixing of the two enabled tight binding of positively charged Ebs to negatively charged dZnONPs. The zeta-potential of dZnONPs, which was originally negative, increased after loading Ebs (Figure S1B). The FTIR results revealed characteristic peaks at 1600 cm^−1^ and 476 cm^−1^, respectively, corresponding to the stretching vibration of the benzene ring in ebselen and the stretching vibration of Zn-O. Both can be observed in Ebs@dZnONPs, indicating that Ebs were successfully loaded onto the dZnONPs (Figure 2B).

The prepared dendritic ZnO nanoparticles were used as a carrier for ebselen and acted on wound healing, the results showed a drug loading rate of 14.96 ± 1.52% (Table S1).

### 2.2. Physicochemical Properties of HGT Hydrogel

The rapid prototyping properties of photocrosslinked hydrogels are ideal for wound repair of various shapes. In order to prepare photocrosslinked hydrogels, in this study, the double bond of acrylate with high reactivity was introduced into the branched hyaluronic acid via a reaction between the epoxy group of glycidyl methacrylate and the carboxyl group of sodium hyaluronate. The ^1^H-NMR spectra showed that (Figure 2C) absorption peaks of HA were detected at 1.9 ppm (COCH_3_, proton d), 3.3~3.9 ppm, and 4.4~4.6 ppm. The new absorption peaks at 6.1, 5.6, and 1.85 ppm corresponded to the two protons of CH_2_ = C (a and b) and the proton of C-CH_3_ (c) on glycidyl methacrylate, respectively. This indicated that we successfully introduced glycidyl methacrylate into HA and obtained acylated hyaluronic acid (HA-GMA).

Designing a suitable photocrosslinked hydrogel network can not only ensure its excellent physical and chemical properties but also provide a better carrier for drug loading. The hydrogel changed from a solution state to a gel state under UV light irradiation, and the cross-section of the hydrogel was observed by SEM to show a porous structure (Figure 2D). Compared to the HG hydrogel, the pores of the HGT hydrogel were denser, with smaller pore sizes from 140.30 ± 76.43 μm to 84.98 ± 35.70 μm (Figure 2E). This is because of the intermolecular interactions between the phenolic hydroxyl groups in TA and the carbonyl groups in HA-GMA, forming a complicated random network of physical bonds, resulting in a double network structure of HGT hydrogels. The HG hydrogel cracked after a certain force was applied externally (Figure 2F). The maximum stresses that HG and HGT hydrogels can withstand are 0.13 MPa and 0.68 MPa, respectively. The maximum stress that HGT can withstand is much greater than HG hydrogel, while the maximum strain that HGT hydrogel can withstand is 70%, while HG hydrogel can only withstand 56% (Figure 2G). After 10 compression cycles, there was a significant loss of HG hydrogel, while the HGT hydrogel was able to maintain a good elastic modulus (Figure 2H). The HGT hydrogel was able to be repeatedly compressed (Video S1), with better fatigue resistance. This is because the addition of tannic acid transforms the rigid and brittle network structure formed by the HG hydrogel into a soft ductile gel network, which significantly improves the mechanical properties of the hydrogel.

### 2.3. Basic Properties of Ebs@ZnONPs/HGT Hydrogel

**Figure 3:**
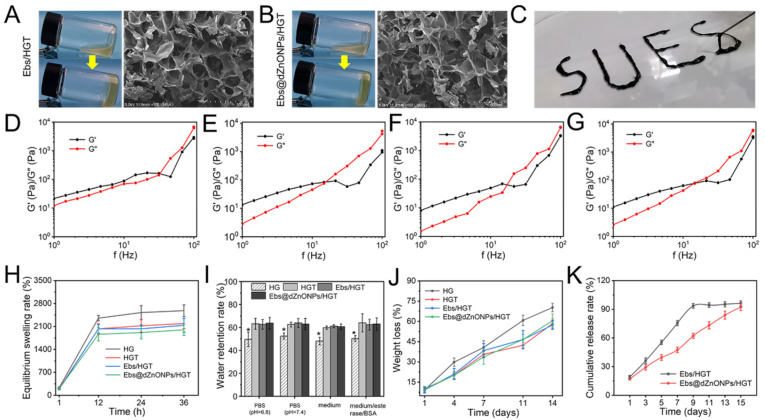
(**A**) Macroscopic image and cross-sectional SEM of Ebs/HGT hydrogel; (**B**) Macroscopic image and cross-sectional SEM of Ebs@dZnONPs/HGT hydrogel; (**C**) Injectability of Ebs@dZnONPs/HGT hydrogel; Frequency sweep testing of HG (**D**), HGT (**E**), Ebs/HGT (**F**) and Ebs@dZnONPs/HGT (**G**) hydrogels; (**H**) Equilibrium swelling of hydrogels in PBS solution (pH = 7.4); (**I**) Water retention of hydrogels after 12 h; (**J**) In vitro degradation of hydrogels; (**K**) In vitro release of Ebs. Data are presented as mean ± standard (*n* = 3), * means *p* < 0.05.

Ebs and Ebs@dZnONPs were doped into HGT hydrogels, respectively. We found that the gelation time of the Ebs@dZnONPs/HGT group was longer than those of the other three groups (Table 1), which may be attributed to the absorption of UV light by ZnO. The internal microscopic morphology of the hydrogels was visualized by SEM (Figure 3A,B). The results showed that the addition of Ebs or Ebs@dZnONPs did not change the microstructure of the HGT hydrogels. The hydrogel presents a connected porous structure with a high specific surface area, which contributed to the storage of water and the efficient diffusion of nutrients and oxygen.

Injectable hydrogel is suitable for the various shapes of a wound, so it can be used as an ideal dressing for wound treatment [36]. The Ebs@dZnONPs/HGT hydrogel could be injected with the word “SUES” written through the syringe (Figure 3C), indicating that the hydrogel had excellent injectability. Rheological properties of the Ebs@dZnONPs/HGT hydrogels were investigated by evaluating their rheological parameters (Figure 3D–G). At low frequencies (Hz), the elastic modulus (G′) was higher than the viscous modulus (G′′), signifying that the hydrogels were elastic materials. As the frequency increased, both G′ and G′′ increased and hydrogels exhibited frequency dependent viscoelastic behavior, consistent with the excellent injectable property of hydrogels.

Bacterial infections can result in an increase in wound exudates. Exudates can soak the surrounding normal tissue [37], while promoting the proliferation of bacteria, potentially resulting in life-threatening, systemic infections [38,39]. The water absorption of hydrogels and their internal structural characteristics were reflected by equilibrium swelling experiments [40]. After 24 h, all four hydrogels had reached equilibrium swelling in weak alkaline solutions (PBS, pH = 7.4) and had an ESR of up to 1900% or more (Figure 3H), enabling rapid water absorption. The same results were obtained in weakly acidic solutions, media, and media with enzymes plus BSA (Figure S2A–C). The results show that the equilibrium swelling rate decreases with the addition of TA. This is because the TA-containing hydrogels have a more compact pore structure, which can effectively reduce the penetration of water molecules [41]. Compared with the HG hydrogel, the hydrogels containing TA could retain about 60% of the water after 12 h (Figure 3I) and about 40% of the water after 24 h (Figure S2D), showing better water retention (*p* * < 0.05). This is also attributed to the denser structure of the double-networked HGT hydrogel, which reduces water loss, and is consistent with the results of the equilibrium swelling experiments.

The biodegradation rate of hydrogels is another significant indicator to assess the availability of wound dressings. The weight of the hydrogels in each group reduced gradually with time, and about 60% weight loss of all hydrogels was observed after 14 days (Figure 3J). Compared with the other three groups, the HG hydrogels showed a faster degradation behavior, which was attributed to the more loose network structure of HG hydrogels and the wider range of motion of water molecules. As the hydrogels degraded, Ebs could be gradually released. In the first five days, approximately 55% of Ebs were released from the Ebs/HGT hydrogel, while about 39% of Ebs were released from the Ebs@dZnONPs/HGT hydrogel (Figure 3K). It could be seen that the release of Ebs slowed down significantly over time. Furthermore, Ebs needed to be released from Ebs@dZnONPs in Ebs@dZnONPs/HGT hydrogels, which slowed down the release rate.

### 2.4. Antimicrobial Properties In Vitro

**Figure 4:**
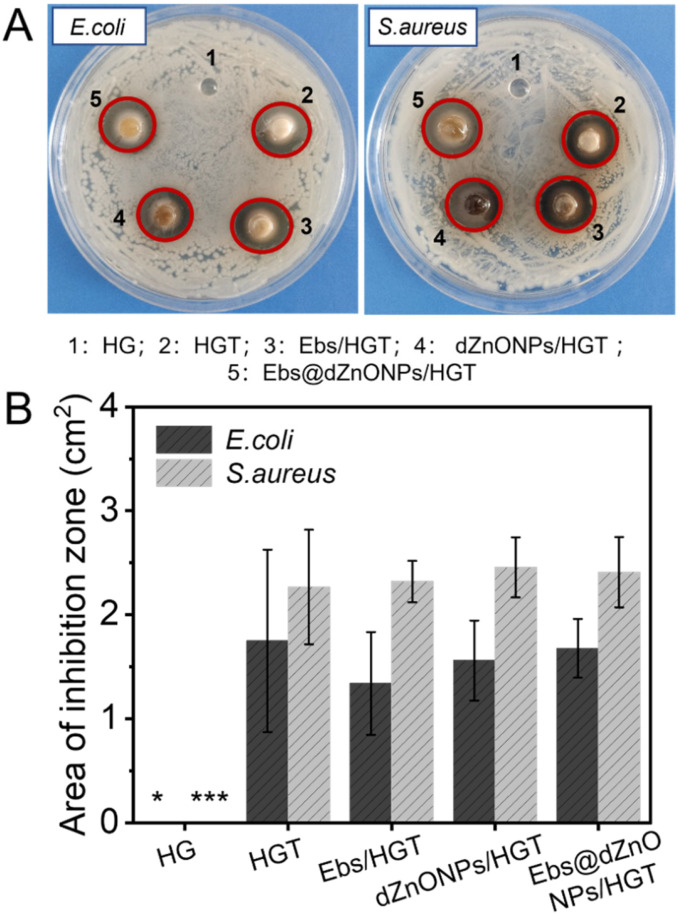
(**A**) Bacteriostatic zone testing of hydrogels; (**B**) Quantitative analysis of the zone of inhibition. Data are presented as mean ± standard (*n* = 3), * means *p* < 0.05, *** means *p* < 0.001.

Without the protection of the skin, the wound is vulnerable to bacterial invasion, which will lead to infection, leading to a variety of complications. We prepared Ebs@dZnONPs/HGT hydrogels with efficient antibacterial activity by doping ebselen and ZnONPs in the hydrogels and validated the antibacterial effect of the hydrogels against gram-negative and gram-positive bacteria through different antibacterial experiments. Firstly, the agar disc diffusion method was used to evaluate the antibacterial activity of hydrogels qualitatively. It could be seen that in addition to HG hydrogel, there were inhibition zones around HGT, Ebs/HGT, dZnONPs/HGT, and Ebs@dZnONPs/HGT hydrogels (Figure 4A). The results showed significant differences between the HG hydrogel group and other groups (Figure 4B). This indicates that the HG hydrogel can not inhibit the growth of *E. coli* and *S. aureus*, and the addition of TA can endow the hydrogels with antibacterial properties. This is because TA contains a wide number of phenolic hydroxy groups, which inhibit cell wall synthesis [42,43] and perturb the potential and biological functions of the cell membrane [44].

**Figure 5:**
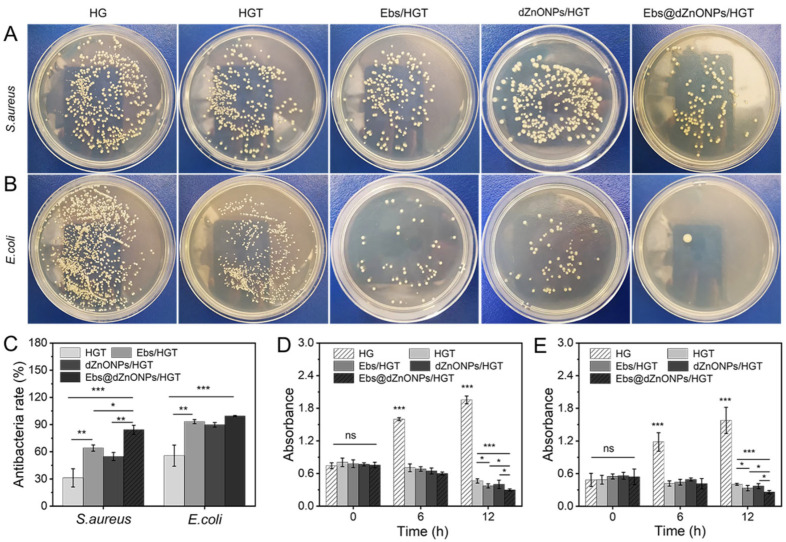
Quantitative analysis of the antibacterial properties of hydrogels: Colony-counting assay of hydrogels against *S. aureus* (**A**) and *E. coli* (**B**); (**C**) Antibacterial rate of hydrogels against *S. aureus* and *E. coli* (quantitative analysis of colony statistics); Quantitative analysis of photometric method against *E. coli* (**D**) and *S. aureus* (**E**). Data are presented as mean ± standard (*n* = 3), * means *p* < 0.05, ** means *p* < 0.01, *** means *p* < 0.001.

Next, the antibacterial activity of hydrogels in each group was quantitatively compared by colony-counting assay. For *S. aureus* and *E. coli* (Figure 5A,B), the bacterial colony numbers of the HG hydrogel group were considerably greater than the other groups. In contrast, the number of colonies was substantially decreased after the addition of TA, Ebs, and dZnONPs. Using the HG hydrogel group as a negative control, the antibacterial rates of HGT, Ebs/HGT and dZnONPs/HGT hydrogels against *E. coli* were 55.77 ± 11.71%, 93.30 ± 2.29%, and 89.74 ± 2.53%, respectively, indicating that the addition of Ebs or ZnO improves the antimicrobial properties of hydrogels. The antibacterial rate of Ebs@dZnONPs/HGT hydrogel increased to 99.43 ± 0.55%, which was stronger than that of Ebs/HGT or dZnONPs/HGT hydrogel and there was a significant difference (* *p* < 0.05), indicating that the combined effect of Ebs and dZnONPs could enhance the antibacterial properties of the hydrogels. The same trend was seen in experiments against *S. aureus* (Figure 5C). This is attributed to the presence of nano-ZnO, which will generate reactive oxygen species to damage the cell membrane. At the same time, the hydrogels will release free Zn ions (Figure S3), and the Zn ions will enter the interior of cells, interfering with cellular metabolic processes and interfering with various enzymes, thus causing them to lose their proper biological function. Ebselen exhibits strong antibacterial activity by disrupting redox homeostasis, achieving the purpose of sterilization [45]. The results indicate that Ebs in combination with dZnONPs has an enhanced antibacterial effect. The results of the photometric method showed that the absorbance of the HG hydrogel group was the highest among all groups and showed a continuously increasing trend. The absorbance of the HGT, Ebs/HGT, dZnONPs/HGT, and Ebs@dZnONPs/HGT hydrogel groups was significantly lower compared to the initial values (Figure 5D,E), and the Ebs@dZnONPs/HGT group had a lower absorbance than the Ebs/HGT and dZnONPs/HGT groups, again verifying the antibacterial potential of Ebs in combination with dZnONPs.

### 2.5. Cytocompatibility

**Figure 6:**
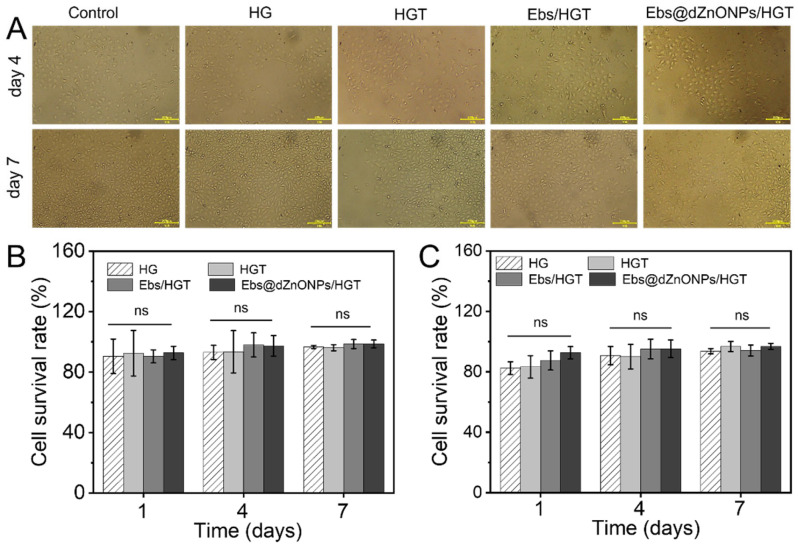
Cytocompatibility of hydrogels. (**A**) Morphology of HUVECs on day 4 and day 7; (**B**) Survival rates of HUVECs on days 1, 4, and 7; (**C**) Survival rates of L929 cells on days 1, 4, and 7. Data are presented as mean ± standard (*n* = 3).

The cytotoxicity of hydrogels was measured by L929 cells and human umbilical vein endothelial cells (HUVECs) cultured in vitro. The microscope image of HUVECs on days four and seven (Figure 6A) showed that HUVECs in each group attached well to the culture dish with regular morphology. Additionally, the cell survival rate of both cells in each group was more than 80% on days one, four, and seven (Figure 6B,C), and no significant differences were found among the hydrogel groups. These results indicate that Ebs@dZnONPs/HGT hydrogel has no significant cytotoxicity, confirming the biosafety of dZnONPs combined with Ebs. In fact, dZnONPs have been reported to have a definite dose-dependent toxicity on cells and affect cell proliferation [27]. While Ebs@dZnONPs/HGT hydrogel did not exhibit cytotoxicity, this might be due to the unique antioxidant properties of Ebs.

### 2.6. Wound Healing Capacity of Hydrogels

**Figure 7:**
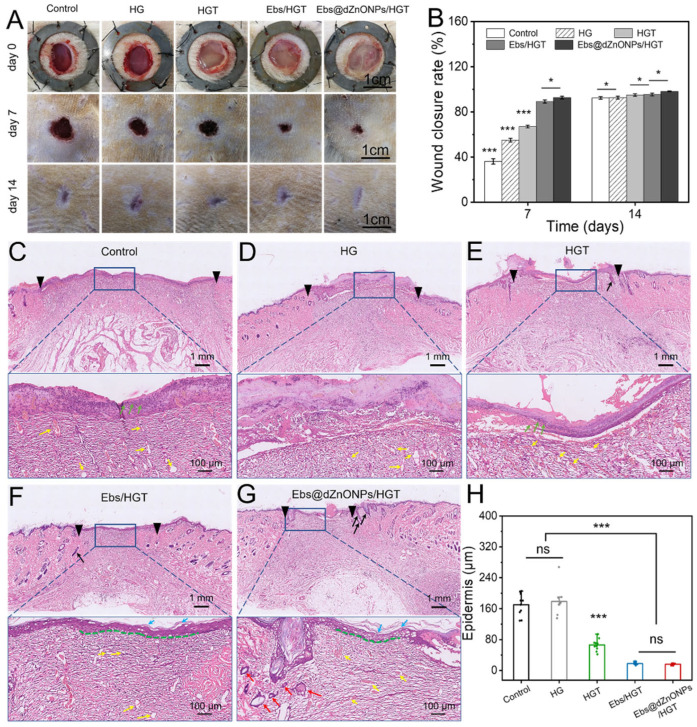
In vivo experiments of hydrogels. (**A**) Macroscopic images of wound healing on days 0, 7, and 14 of treatment; (**B**) Wound closure rates on days 7 and 14 of treatment. Results of HE staining of the new skin after 7 days of treatment: (**C**) Blank group, (**D**) HG hydrogel group, (**E**) HGT hydrogel group, (**F**) Ebs/HGT hydrogel group, (**G**) Ebs@dZnONPs/HGT hydrogel group, (**H**) Quantitative analysis of epidermal layer thickness. Data are presented as mean ± standard (*n* = 3), * means *p* < 0.05, *** means *p* < 0.001.

The efficacy of Ebs@dZnONPs/HGT hydrogel on wound healing was evaluated using a rat full-thickness infected cutaneous wound model. Figure S4 shows that the infectious cutaneous wound model was successfully constructed. The wound situation and wound size in different treatment groups on days 0, 7, and 14 were recorded (Figure 7A). On postoperative day 7, the wound area was significantly smaller in hydrogel treated groups than that in the blank group (Figure 7B). Among them, the healing rate of the HG group was 55.04 ± 1.59%, which exceeded the rate obtained for the blank group, 36.20 ± 2.0% (*** *p* < 0.001), indicating that the HG hydrogel had a therapeutic benefit for infected skin wound healing. The healing rates of HGT, Ebs/HGT, and Ebs@dZnONPs/HGT groups, respectively, were 67.02 ± 1.23%, 89.18 ± 1.37%, and 92.61 ± 1.17%. On postoperative day 14, the scar of the Ebs@dZnONPs/HGT group was minimal with the lightest scar color in comparison to other groups. These results suggest that the addition of ebselen with antibacterial and antioxidant characteristics contributes to wound healing, and the combination of dZnONPs and Ebs can further enhance the therapeutic effect of hydrogel on infected wounds.

On postoperative day 7, wound tissues in each group were taken for H&E staining to further evaluate the quality of wound healing (Figure 7C–G). It could be seen that there were obvious differences in the wound healing qualities among all groups. The wound distance in the blank group was significantly wider than that in other groups. Besides, epithelialization was also incomplete. There were also a large number of infiltrated inflammatory cells (green arrows) in the wound area. When compared to the blank group, the groups having the wound covered by HG or HGT hydrogel presented a better wound healing quality. We could see that the wound distance was smaller. Even though epithelialization was discontinuous, the stratum corneum and stratum basalis were still visible. This is due to the three-dimensional porous structure of hydrogels, which enables the exchange of gases and the circulation of nutrients while absorbing excess exudates. In addition, TA also has some antibacterial activity. All those factors played a role in the wound healing process. The wound distances in Ebs/HGT and Ebs@dZnONPs/HGT groups were significantly smaller than in the other groups. The epithelial structure was continuous with intact stratum corneum (light blue arrows) and stratum basalis (green dotted line). In the Ebs@dZnONPs/HGT group, the number of capillaries (yellow arrows) in the wound area decreased and more mature small blood vessels (red arrows) appeared. The most significant aspect was the regeneration of hair follicles (black arrows) in the dermis area. Furthermore, quantification of epidermal thickness revealed that the epidermal layers in the blank and HG groups were obviously thicker with scabs covering the wound surface compared with the other groups (Figure 7H). Conversely, wounds in the Ebs/HGT and Ebs@dZnONPs/HGT groups possessed a flat surface and uniform thickness, presenting more advanced stages of epithelial maturation. This result demonstrates that the Ebs@dZnONPs/HGT hydrogel can improve re-epithelialization in infected wounds.

**Figure 8:**
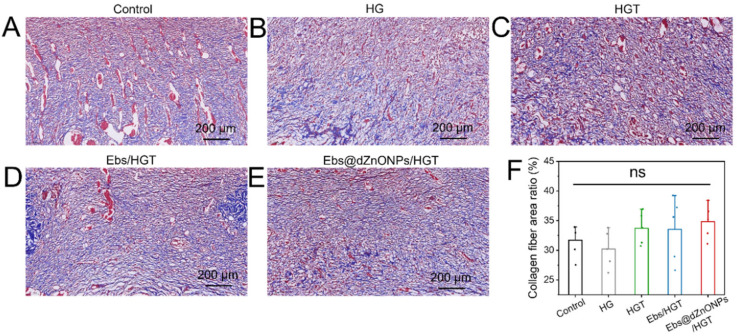
Results of Masson’s trichrome staining after 7 days of treatment. (**A**) Blank group, (**B**) HG hydrogel group, (**C**) HGT hydrogel group, (**D**) Ebs/HGT hydrogel, (**E**) Ebs@dZnONPs/HGT hydrogel, (**F**) Quantitative analysis of collagen deposition area. Data are presented as mean ± standard (*n* = 3).

Meanwhile, the collagen deposition on day 7 was evaluated by Masson’s trichrome stain, and the deposited collagen was stained blue. It could be seen that compared with the other groups, the Ebs, Ebs/HGT and Ebs@dZnONPs/HGT groups had more collagen deposition with a more uniform arrangement (Figure 8A–E). However, quantitative analysis of the collagen depositing area revealed no statistically significant difference among all groups (Figure 8F), indicating that the combination of ebselen and dZnONPs might slightly promote collagen deposition.

Additionally, the skin surface of rats in each group showed no signs of erythema, edema, or irritation during wound healing, indicating that hydrogels had no local damage. In conclusion, these results demonstrate that the Ebs@dZnONPs/HGT hydrogel can improve re-epithelialization, and significantly accelerate wound healing with excellent biocompatibility.

## 3. Conclusions

In summary, we prepared a photocrosslinked Ebs@dZnONPs/HGT hydrogel wound dressing with antibacterial and excellent biosafety. Hydrogel also has good water absorption, water retention, and injectable and biodegradable properties and can accelerate the healing of infectious wounds. Therefore, Ebs@dZnONPs/HGT hydrogel has broad application prospects. To our knowledge, this is the first combined use of Ebs and ZnONPs for wound healing. Loading Ebs on dZnONPs can delay the release of Ebs, and the combination of Ebs and dZnONPs can kill bacteria more effectively without obvious cytotoxicity. The combination of Ebs and ZnONPs has provided novel insights for the treatment of infectious wounds and even more infectious diseases.

## 4. Materials and Methods

### 4.1. Materials

Ebselen (Ebs, purity ≥ 98%) was collected from Shanghai Kemin Biotechnology Co., Ltd. (Shanghai, China). Sodium hyaluronate (HA, Mw = 1.5 × 10^6^) was collected from Shanghai yuanye Bio-Technology Co., Ltd. (Shanghai, China). Glycidyl methacrylate (GMA, purity ≥ 99%), glycine (Gly, purity ≥ 99%), cyclohexane (Cy, purity ≥ 99.9%), cetyl trimethyl ammonium bromide (CTAB, purity ≥ 99%), and triethyl phosphate (TEP, purity ≥ 99%) were collected from Adamas Reagent Co., Ltd. (Shanghai, China). Ethyl orthosilicate (TEOS, SiO_2_ ≥ 28.4%), and Zn (NO_3_)_2_·6 H_2_O was collected from Sinopharm Chemical Reagent Co., Ltd. (Shanghai, China). Mouse fibroblasts cells (L929) for experiments in vitro were obtained from Shanghai Cell Bank of Chinese Academy of Sciences (Shanghai, China). In addition, Sigma-Aldrich Trading Co., Ltd. (Shanghai, China) provided the Cell Counting Kit-8 (CCK-8) for this study. Dulbecco’s modified eagle medium (DMEM), Fetal bovine serum (FBS), and double antibody (penicillin/streptomycin) were obtained from Hyclone Trading Co., Ltd. (Tianjin, China). Unless otherwise indicated, all the above reagents were used directly. All the materials were used as received, except where mentioned otherwise.

### 4.2. Synthesis of dZnONPs and Ebs@dZnONP

The dZnONPs were prepared by the epitaxial growth method [46]. In a round-bottom flask, CTAB (1.0 g) and NaOH (0.8 mL, 0.1 M) were added to 50 mL water and gently stirred for 2 h at 60 °C, then 2.53 g Zn (NO_3_)_2_·6 H_2_O was added to this solution and continuously stirred for 0.5 h. Afterward, 0.4092 mL TEP was added to the above solution and stirred magnetically for 0.5 h, then 4 mL TEOS and 16 mL cyclohexane were added and kept under magnetic churning for 48 h at 60 °C. The products were collected by centrifugation, rinsed multiple times with water and ethanol, centrifuged again and freeze-dried. After high-temperature calcination in a muffle furnace (650 °C) for 3 h, dZnONPs were obtained.

The dZnONPs and Ebs were dispersed in anhydrous ethanol at the mass ratio of 1:1, and then churned at low speed for 24 h and centrifuged (10,000 rpm/min, 10 min). The Ebs-loaded dZnONPs were collected and referred to as Ebs@dZnONPs. The absorbance values of Ebs at 225 nm were tested using a UV-Vis spectrophotometer. According to the standard curve, the drug loading rate was calculated by Equation (1):

(1) $$\mathrm{drug}\;\mathrm{loading}\;\mathrm{rate}=\frac{m_{0\;}{-\;m}_{1}}{m}\;\times \;100\%\;$$

where m_0_ stands for the initial mass of Ebs, m_1_ is the amount of Ebs in supernatant and m stands for the amount of dZnONPs.

### 4.3. Acylation of HA

Glycidyl methacrylate was grafted to HA to acylate the HA [47]. 1 g HA was dissolved in 100 mL distilled water, then 3.2 mL glycidyl methacrylate (GMA) was added under constant stirring. The mixture was kept stirring at 50 °C for 24 h. To terminate the reaction, 4 mL glycine (20% *w*/*v*) solution was added and stirred at room temperature for 30 min. Afterward, the sample was lyophilized after being dialyzed (MWCO = 3000 Da) in water. The final product was acylated hyaluronic acid (HA-GMA).

### 4.4. Synthesis of Ebs@dZnONPs/HGT Hydrogels

Acylated hyaluronic acid (HA-GMA, 3%, *w*/*v*) and tannic acid (TA, 4%, *w*/*v*) were co-mixed. Afterward, Ebs@dZnONPs was added to the co-mixed solution at a concentration of 2 mg/mL and stirred until it was uniformly dispersed. Then, photoinitiator I2959 (1%) was added and mixed well. Finally, Ebs@dZnONPs/HGT hydrogels were obtained by UV illumination. HG hydrogels, HGT hydrogels, Ebs/HGT hydrogels, and dZnONPs/HGT hydrogels were prepared according to Table 1, and the gelation times mentioned in the table are based on 1 mL of solution.

### 4.5. Characterization and Testing of dZnONPs, Acylated Hyaluronic Acid, and Hydrogel

The transmission electron microscope (TEM) was used to analyze the morphology of the dZnONPs, and the Image-J was used to assess the particle size distribution of the dZnONPs. The Zeta potentials of Ebs, dZnONPs and Ebs@dZnONPs were tested by zeta potential analyzer (Dynamic Light Scattering, DLS). Then, Ebs, dZnONPs, and Ebs@dZnONPs were characterized by Fourier Transform Infrared Spectrometer (FTIR). HA-GMA and HA were characterized by ^1^H-NMR using D_2_O as solvent. The cross-sectional structure of the hydrogel was visualized by scanning electron microscope (SEM).

The mechanical characteristics of hydrogels in each group were measured using a high-precision tensile testing machine (HY-025CS) with a load range of 0~200 N at room temperature strictly according to ISO 7198:1998. Each sample was prepared as a cylinder (8.0 mm in diameter and 5.0 mm in height). The compression tests (0.2 mm/min) were performed on the prepared samples until cracking. At the same time, stress-strain curves were recorded and ultimate stress was evaluated. Furthermore, the samples were subjected to a cyclic compression test with 30% deformation (10 times, 0.4 mm/min). The force-time curves were used to observe the force lost during the task.

The rheology of the hydrogels was analyzed with a rotational rheometer (HAAKE, Thermo Fisher Scientific Co., Ltd. Shanghai, China). Their G′ and G′′ were tested as the frequency was increased from 1 Hz to 100 Hz. The injectability of the hydrogels was evaluated by syringes. The water absorption property of the hydrogels was evaluated by equilibrium swelling experiment, and the water retention capacity of the hydrogels was determined after a period of time. For detailed procedures, see the Supporting Information (SI).

### 4.6. Degradation of Hydrogels and Release of Ebs In Vitro

In brief, HG, HGT, Ebs/HGT and Ebs@dZnONPs/HGT hydrogels were lyophilized, subsequently weighed to record the initial mass, and then soaked in PBS at pH = 7.4. At different time points, hydrogels were removed from PBS, lyophilized, and weighed. The weight loss rate of the hydrogels in each group was determined by Equation (2):

(2) $$\mathrm{weight}\;\mathrm{loss}\;\mathrm{rate}=\frac{W_{0\;}{-\;W}_{t}}{W_{0}}\;\times \;100\%$$

where W_0_ stands for the initial mass of hydrogel after freeze-drying and W_t_ is the mass of hydrogels after freeze-drying at different time points.

The Ebs@dZnONPs/HGT hydrogels were placed in 20 mL PBS (pH = 7.4) buffer and shook on a constant-temperature shaker. The concentration of zinc ions was measured by Inductively coupled plasma mass spectrometry (ICP-MS) at different time points and recorded. The Ebs/HGT and Ebs@dZnONPs/HGT hydrogels were put into 10 mL PBS (pH = 7.4) buffer. At different time points, 10 mL of the solution was taken out and centrifuged (10,000 rap/min, 8 min). The supernatant was then discarded and anhydrous ethanol was added. Absorbance values were measured at 225 nm by a UV-Vis spectrophotometer. The concentration of Ebs was calculated from the linear regression equation of the standard curve. The cumulative release rate was calculated from Equation (3):

(3) $$\mathrm{cumulative}\;\mathrm{release}\;\mathrm{rate}=\frac{\sum \frac{C}{1000}\;\times \;V}{m_{d}}\;\times \;100\%$$

where C is the concentration of Ebs (mg/L), V stands for the volume of the solution (mL), and m_d_ is the total mass of Ebs contained in the hydrogel (mg).

### 4.7. Antibacterial Activity and Cell Culture In Vitro

The antibacterial effect of the hydrogels in each group against *E. coli* and *S. aureus* was assessed by the agar disc diffusion way, colony-counting assay, and the photometric method. In brief, after bacteria were cultured overnight in LB broth, 20 μL of bacteria solution was then spread evenly on the surface of a solid medium for use in agar disc diffusion experiments. In addition, a certain amount of bacteria solution was placed in a sterile centrifuge tube, and then, an equal amount of hydrogel from each group was added. These samples were used for colony-counting assays and photometric experiments. The detailed experimental steps are shown in the Supporting Information (SI).

Mouse fibroblasts (L929) and HUVECs were cultured in complete medium (DMEM containing 10% FBS and 1% penicillin-streptomycin). HG, HGT, Ebs/HGT, and Ebs@dZnONPs/HGT hydrogels were sterilized by 75% alcohol and ultraviolet irradiation and then immersed in medium (0.1 g/mL) to obtain the leach liquor. L929 cells were evenly seeded in a 24-well plate (1.0 × 10^4^ cells/well) and cultured with the leach liquor mentioned above. The leach liquor was replaced every two days. After 1, 4, and 7 days of incubation, the OD value was measured at 450 nm, and then the cell survival rate was calculated by Equation (4). In addition, the cell morphology of HUVECs was observed using an inverted fluorescence microscope on days 4 and 7.

(4) $$\mathrm{cell}\;\mathrm{survival}\;\mathrm{rate}=\frac{OD-OD_{cck}}{OD_{0}-OD_{CCK}}\;\times \;100\%$$

where *OD* is the optical density value of cells in the experimental group, *OD_cck_* stands for the optical density value of cck-8, and *OD*_0_ is the optical density value of the control group.

### 4.8. Wound Healing Assessment In Vivo

All animals were from Shanghai Jiesijie Laboratory Animal Co., Ltd (Shanghai, China) and all experimental procedures were performed in accordance with the Shanghai Sixth People’s Hospital’s Animal Welfare Ethics Committee’s guidelines (Shanghai, China). Sprague-Dawley (SD) rats (male, about 160 g) were used to establish the skin infection model. The rat dorsum was shaved and disinfected to create a full-thickness skin defect measuring 1 cm in diameter. Then, 500 µL of the mixed bacterial solution (absorbance of 1.7995) of *E. coli* and *S. aureus* was inoculated to the skin wound and infected for 3 h. The tissue fluid around the wound surface was collected, smeared uniformly on the agar plates, and incubated overnight. The wounds in each group were covered with sterilized gauze, HG, HGT, Ebs/HGT, or Ebs@dZnONPs/HGT hydrogels, respectively. On days 7 and 14 after surgery, digital photographs of wounds were taken to assess wound healing. The wound closure rate was calculated using Equation (5):

(5) $$\mathrm{wound}\;\mathrm{closure}\;\mathrm{rate}=\frac{S_{0\;}{-\;S}_{t}}{S_{0}}\;\times \;100\%\;$$

where S_0_ stands for the initial wound area and S_t_ is the wound area at different time points.

### 4.9. Histological Staining

On day 7 after treatment, the rats were euthanized and skin samples were obtained for further analysis. Fresh skin samples were fixed in paraformaldehyde solution, dehydrated, embedded in paraffin, sectioned, dewaxed, and rehydrated. They were then stained with hematoxylin and eosin (H&E) and Masson, respectively. The morphological change and collagen fiber deposition in wound tissue were observed by optical microscopy.

### 4.10. Statistical Analysis

Statistical analysis was performed by SPSS software (IBM, Armonk, NY, USA) using one-way analysis of variance (ANOVA). Mean ± standard deviation (Mean ± SD) was used to express all quantitative data. *p*-value < 0.05 (*) was regarded as being statistically significant. * indicates *p* < 0.05, ** indicates *p* < 0.01, and *** indicates *p* < 0.001.

## Figures and Tables

**Table 1:** The preparation parameters of hydrogels for HG, HGT, Ebs/HGT, dZnONPs/HGT and Ebs@dZnONPs/HGT, respectively.

Samples	HA-GMA (*w*/*v*%)	TA (*w*/*v*%)	Ebs/dZnONPs (mg/mL)	Ebs@dZnONPs (mg/mL)	Gelation Time (min)
HG	3.0	0.0	0.0/0.0	0.0	<2
HGT	3.0	4.0	0.0/0.0	0.0	<2
Ebs/HGT	3.0	4.0	2.0/0.0	0.0	<2
dZnONPs/HGT	3.0	4.0	0.0/2.0	0.0	2~5
Ebs@dZnONPs/HGT	3.0	4.0	0.0/0.0	2.0	2~5

## Data Availability

Not applicable.

## Supplementary Materials

The following supporting information can be downloaded at: https://www.mdpi.com/article/10.3390/gels8080463/s1, Figure S1: (A) Particle size distribution of dZnONPs; (B) Zeta potential of dZnONPs, Ebs and Ebs@dZnONPs; Figure S2: Equilibrium swelling of hydrogels in (A) PBS solution (pH = 6.8); (B) medium solution; (C) medium/esterase/BSA solution. (D) Water retention of hydrogels after 24 h; Figure S3: Release profiles of zinc ions (dissociated from ZnONPs) in hydrogels, Figure S4: Plate smearing test using tissue fluid around the wound after infection in each group; Table S1: Drug loading rate of ebselen; Video S1: HGT hydrogel compression resilience.
