# Correction: Chen et al. A dZnONPs Enhanced Hybrid Injectable Photocrosslinked Hydrogel for Infected Wounds Treatment. *Gels* 2022, *8*, 463

**DOI:** 10.3390/gels12070579

**Published:** 2026-06-30

**Authors:** Yao Chen, Yu Xiang, Tonghe Zhu, Sihao Chen, Juan Du, Jiajia Luo, Xiaoyu Yan

**Affiliations:** 1 School of Chemistry and Chemical Engineering, Shanghai Engineering Research Center of Pharmaceutical Intelligent Equipment, Shanghai Frontiers Science Research Center for Druggability of Cardiovascular Non-Coding RNA, Institute for Frontier Medical Technology, Shanghai University of Engineering Science, 333 Longteng Rd., Shanghai 201620, China; m040119408@sues.edu.cn (Y.C.); chensh@sues.edu.cn (S.C.); bairuochen12@163.com (J.D.);; 2 Department of Sports Medicine, Department of Orthopedics, Shanghai Jiao Tong University Affiliated Sixth People’s Hospital, 600 Yishan Rd., Shanghai 200233, China; knight-errant@sjtu.edu.cn

In the original publication [1], there were duplications in the subfigures of Figure 3. The authors have conducted new experiments, and the corrected Figure 3 is shown below. The authors state that the scientific conclusions are unaffected. This correction was approved by the Academic Editor. The original publication has also been updated.

## Figures and Tables

**Figure 3:**
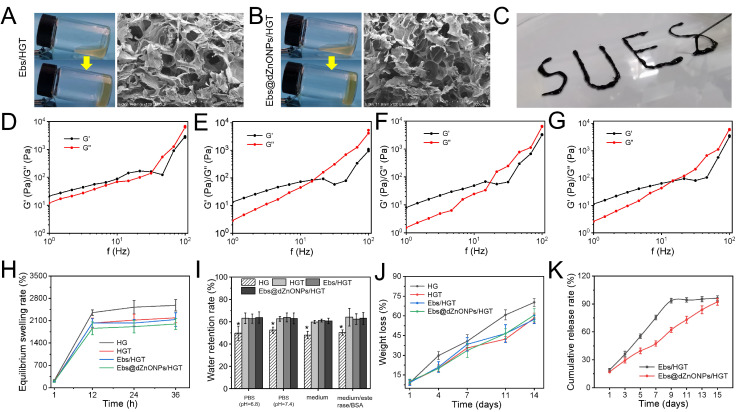
(**A**) Macroscopic image and cross-sectional SEM of Ebs/HGT hydrogel; (**B**) macroscopic image and cross-sectional SEM of Ebs@dZnONP/HGT hydrogel; (**C**) injectability of Ebs@dZnONP/HGT hydrogel; frequency sweep testing of HG (**D**), HGT (**E**), Ebs/HGT (**F**) and Ebs@dZnONP/HGT (**G**) hydrogels; (**H**) equilibrium swelling of hydrogels; (**I**) water retention of hydrogels; (**J**) in vitro degradation of hydrogels; (**K**) in vitro release of Ebs. Data are presented as mean ± standard deviation (*n* = 3); * means *p* < 0.05.
