# Supplementary material for: A dZnONPs Enhanced Hybrid Injectable Photocrosslinked Hydrogel for Infected Wounds Treatment
Source: Gels. 2022 Jul 24;8(8):463. doi: 10.3390/gels8080463 (PMC9329969; doi:10.3390/gels8080463)
Supplement: Supplementary file 1 [file gels-08-00463-s001.zip › gels-1799193-supplementary.pdf]

Supplementary Materials

Materials and methods

Injectability testing of Ebs@dZnONPs/HGT hydrogels:

The Ebs@dZnONPs/HGT hydrogels were pre-crosslinked by UV light for 30~60 seconds before being stained with black ink (to visually observe the changes of the hydrogels). The pre-crosslinked hydrogels were loaded into a 1 mL syringe and extruded onto a smooth plate to write "SUES".

Equilibrium swelling rate of hydrogels:

The hydrogels were placed in pH = 6.8 PBS, pH = 7.4 PBS, high sugar medium, or high sugar medium supplemented with esterase and BSA. At predetermined time points (1 h, 12 h, 24 h, and 36 h), the swollen hydrogels were removed and weighed after removal of excess water. The equilibrium swelling rates were determined by Eq. (1).

$$\text{Equilibrium swelling rates} = \frac{m_a - m_b}{m_b} \times 100\% \quad (1)$$

where m_a expresses the mass of the hydrogel after swelling, g; m_b stands for the mass of the Initial hydrogel, g.

Water retention rate of hydrogels:

The hydrogels were placed in pH = 6.8 PBS, pH = 7.4 PBS, high sugar medium, or high sugar medium with added esterase and BSA for 24 h to achieve the equilibrium swelling state. The swollen hydrogels were then removed from the solutions, rested at room temperature, and weighed at 12 h and 24 h. The water retention rate was determined by Eq. (2).

$$\text{Water retention rate} = \frac{m_c - m_b}{m_a - m_b} \times 100\% \quad (2)$$

where m_a expresses the mass of the hydrogel after swelling, g; m_b stands for the mass of the Initial hydrogel, g; m_c is the mass of the hydrogel after 12 h or 24 h, g.

Antimicrobial testing of hydrogels:

Antibacterial circle test:

The hydrogel was prepared to be a cylindrical shape and sterilised by UV irradiation. The bacteria were transferred to LB broth under aseptic conditions and incubated at a constant temperature in a shaker at 37 °C for 12 h. Then, about 20 μL of the bacterial solution was smeared on the surface of the solid medium evenly. The hydrogels in each group were gently added to the solid medium and the culture dishes were placed in a constant temperature shaker for 12 h. Finally, the zone of inhibition (cm) was calculated using the Image-J software.

Colony statistics:

E. coli (BCRC 11634) and *S. aureus* (BCRC 10451) were transferred to LB broth (liquid) in a sterile environment and incubated for 12 h. The bacterial broth was aspirated and placed in sterile centrifuge tubes. The same amounts of HG, HGT, Ebs/HGT, dZnONPs/HGT and Ebs@dZnONPs/HGT hydrogels were then added to the centrifuge tubes and the centrifuge tubes were placed in a constant temperature shaker at 37 °C for a certain period of time. The co-cultures were diluted 10,000 times and 10 μL of the diluted bacterial solution was smeared on the surface of the solid medium. Finally, the culture dishes were placed in a constant temperature shaker at 37 °C for 12 h and the number of colonies was counted.

Photometric testing:

E. coli (BCRC 11634) and *S. aureus* (BCRC 10451) were transferred to LB broth (liquid) in a

sterile environment and incubated for 12 h. The bacterial broth was aspirated and placed in sterile centrifuge tubes. The same amounts of HG, HGT, Ebs/HGT, dZnONPs/HGT and Ebs@dZnONPs/HGT hydrogels were then added to the centrifuge tubes and the centrifuge tubes were placed in a constant temperature shaker at 37 °C for a certain period of time. The final absorbance (600 nm) of the tubes was recorded and the difference between the initial and final absorbance was determined. The antibacterial properties of the hydrogels were quantified by the difference between the initial and final absorbance

Table S1 Drug loading rate of ebselen.

Ebs:dZnONPs	1: 1
drug loading rate	14.96 ± 1.52%

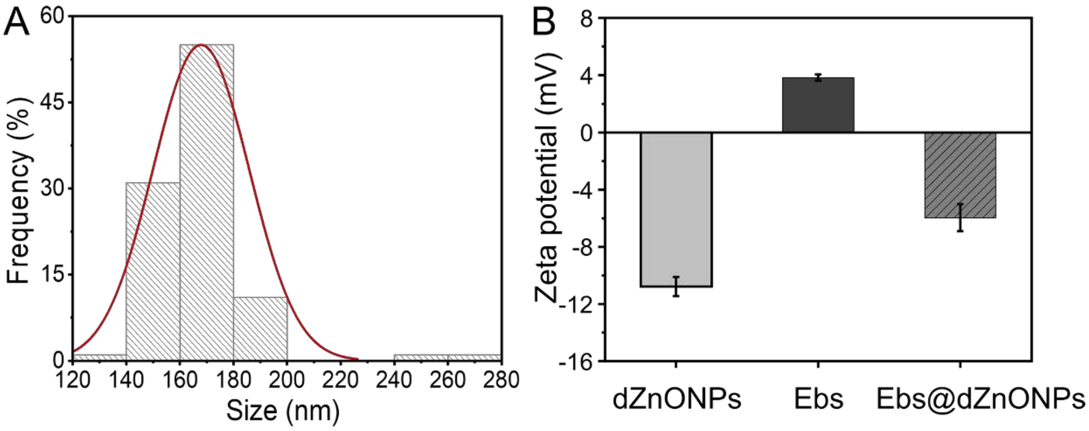

Figure S1. (A) Particle size distribution of dZnONPs; (B) Zeta potential of dZnONPs, Ebs and Ebs@dZnONPs.

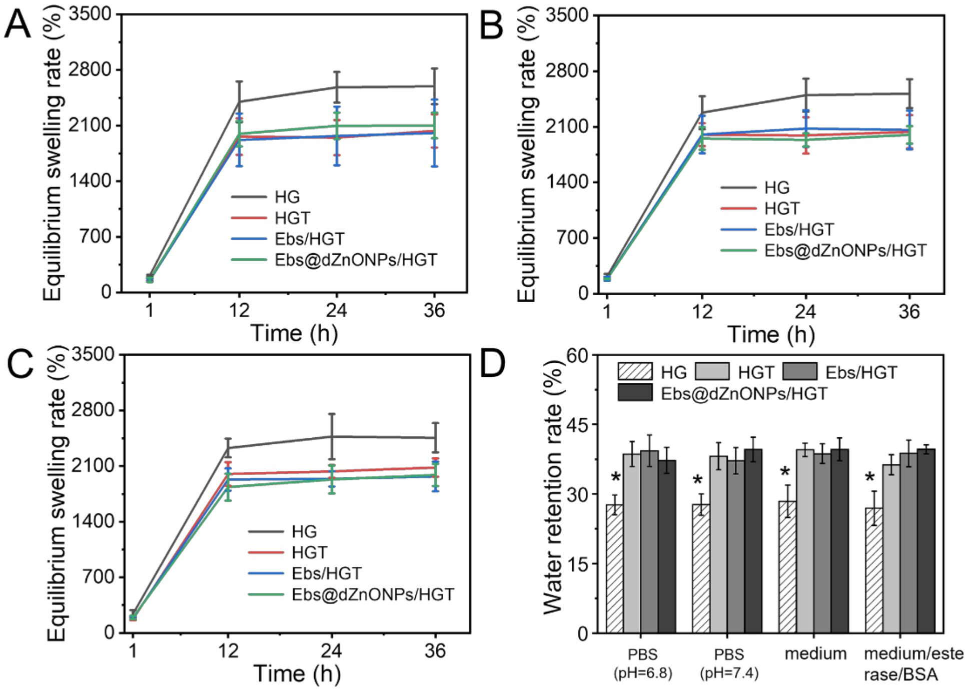

Figure S2. Equilibrium swelling of hydrogels in (A) PBS solution (pH=6.8); (B) medium solution; (C) medium/esterase/BSA solution. (D) Water retention of hydrogels after 24 h. Data are presented as mean ± standard (n=3), * means P < 0.05.

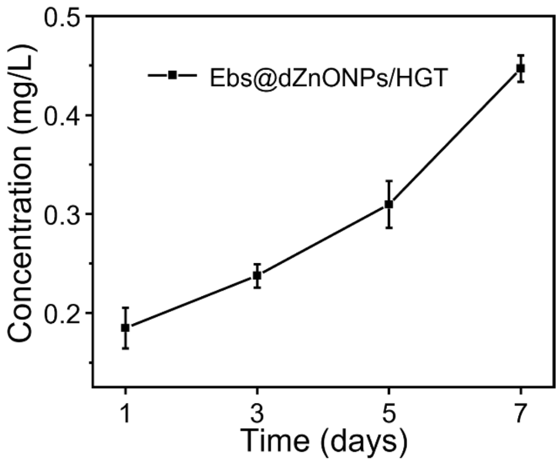

Figure S3. Release profiles of zinc ions (dissociated from ZnONPs) in hydrogels.

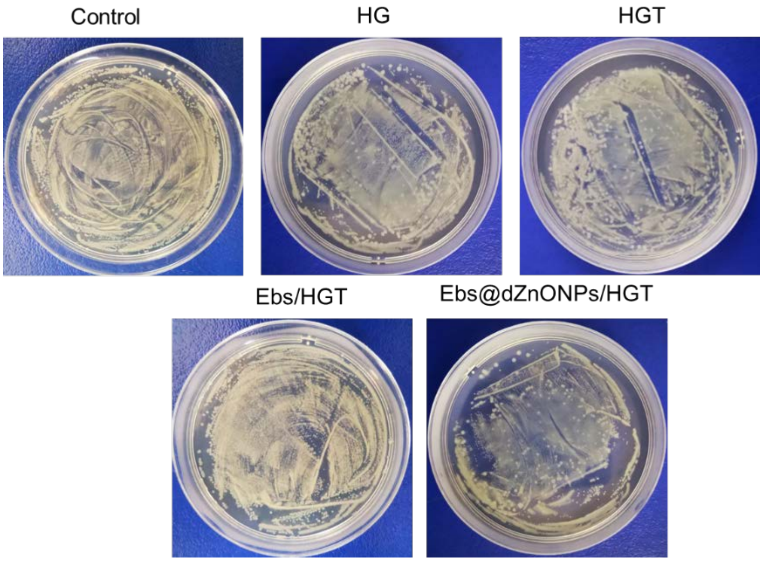

Figure S4. Plate smearing test using tissue fluid around the wound after infection in each group.
